# IMU-838, a Developmental DHODH Inhibitor in Phase II for Autoimmune Disease, Shows Anti-SARS-CoV-2 and Broad-Spectrum Antiviral Efficacy In Vitro

**DOI:** 10.3390/v12121394

**Published:** 2020-12-05

**Authors:** Friedrich Hahn, Christina Wangen, Sigrun Häge, Antonia Sophia Peter, Gerhard Dobler, Brett Hurst, Justin Julander, Jonas Fuchs, Zsolt Ruzsics, Klaus Überla, Hans-Martin Jäck, Roger Ptak, Andreas Muehler, Manfred Gröppel, Daniel Vitt, Evelyn Peelen, Hella Kohlhof, Manfred Marschall

**Affiliations:** 1 Institute for Clinical and Molecular Virology, Friedrich-Alexander University of Erlangen-Nürnberg (FAU), Schlossgarten 4, 91054 Erlangen, Germany; friedrich.hahn@uk-erlangen.de (F.H.); christina.wangen@uk-erlangen.de (C.W.); sigrun.haege@fau.de (S.H.); antoniasophia.peter@uk-erlangen.de (A.S.P.); klaus.ueberla@fau.de (K.Ü.); 2 Institute of Microbiology, Bundeswehr, Neuherbergstraße 11, 80937 München, Germany; gerharddobler@bundeswehr.org; 3 Institute for Antiviral Research, Utah State University, 5600 Old Main Hill, Logan, UT 84322, USA; brett.hurst@usu.edu (B.H.); justin.julander@usu.edu (J.J.); 4 Institute for Virology, University Medical Center/Universitätsklinikum Freiburg, Hermann-Herder Str 11, 79104 Freiburg, Germany; jonas.fuchs@uniklinik-freiburg.de (J.F.); zsolt.ruzsics@uniklinik-freiburg.de (Z.R.); 5 Division of Immunology, Department of Medicine 3, FAU, Glückstraße 6, 91054 Erlangen, Germany; hans-martin.jaeck@fau.de; 6 Drug Development Division, Infectious Disease Research, Southern Research, 431 Aviation Way, Frederick, MD 21701, USA; rptak@southernresearch.org; 7 Immunic AG, Lochhamer Schlag 21, 82166 Gräfelfing, Germany; andreas.muehler@imux.com (A.M.); manfred.groeppel@imux.com (M.G.); daniel.vitt@imux.com (D.V.); evelyn.peelen@imux.com (E.P.); hella.kohlhof@imux.com (H.K.)

**Keywords:** SARS-CoV-2, antiviral therapy, host-directed antivirals (HDAs), dihydroorotate dehydrogenase (DHODH) inhibitors, IMU-838, vidofludimus

## Abstract

The ongoing pandemic spread of the severe acute respiratory syndrome coronavirus type 2 (SARS-CoV-2) demands skillful strategies for novel drug development, drug repurposing and cotreatments, in particular focusing on existing candidates of host-directed antivirals (HDAs). The developmental drug IMU-838, currently being investigated in a phase 2b trial in patients suffering from autoimmune diseases, represents an inhibitor of human dihydroorotate dehydrogenase (DHODH) with a recently proven antiviral activity in vitro and in vivo. Here, we established an analysis system for assessing the antiviral potency of IMU-838 and DHODH-directed back-up drugs in cultured cell-based infection models. By the use of SARS-CoV-2-specific immunofluorescence, Western blot, in-cell ELISA, viral yield reduction and RT-qPCR methods, we demonstrated the following: (i) IMU-838 and back-ups show anti-SARS-CoV-2 activity at several levels of viral replication, i.e., protein production, double-strand RNA synthesis, and release of infectious virus; (ii) antiviral efficacy in Vero cells was demonstrated in a micromolar range (IMU-838 half-maximal effective concentration, EC_50,_ of 7.6 ± 5.8 µM); (iii) anti-SARS-CoV-2 activity was distinct from cytotoxic effects (half-cytotoxic concentration, CC_50,_ >100 µM); (iv) the drug in vitro potency was confirmed using several Vero lineages and human cells; (v) combination with remdesivir showed enhanced anti-SARS-CoV-2 activity; (vi) vidofludimus, the active determinant of IMU-838, exerted a broad-spectrum activity against a selection of major human pathogenic viruses. These findings strongly suggest that developmental DHODH inhibitors represent promising candidates for use as anti-SARS-CoV-2 therapeutics.

## 1. Introduction

Emerging viruses have repeatedly raised challenging medical problems due to the hurdles in rapidly generating test systems, vaccines and antiviral drugs. IMU-838 is a next-generation inhibitor of human dihydroorotate dehydrogenase (DHODH) containing the moiety of vidofludimus as its active determinant. The oral drug formulation is currently in phase 2 clinical development for autoimmune diseases including multiple sclerosis, ulcerative colitis and primary sclerosing cholangitis. More than 650 individuals have already been treated with IMU-838 or its active moiety, and the safety profile is comparable to the placebo cohort [1,2,3]. Data from the EMPhASIS trial have shown activity of IMU-838 in multiple sclerosis patients who have met the primary and secondary endpoints with high statistical significance [3]. DHODH inhibitors are known to inhibit metabolically active cells, such as cancer cells and hyperactivated lymphocytes [4]. A highly relevant improvement in understanding the mechanisms of DHODH inhibitors was the finding that virus-infected cells are similarly metabolically active and, thus, show strict dependence on DHODH for maintaining their high metabolic turnover [5]. In these cells, the extraordinary demand of nucleotides cannot be fulfilled by the pyrimidine salvage pathway. For this reason, the *de novo* synthesis of pyrimidines needs to be activated and sustained at an increased level, a phenomenon of metabolic upregulation similarly detectable upon viral, tumoral or immunological stimuli. Particularly in the case of virus infections, the pharmacological inhibition of activated *de novo* synthesis may result in a block of nucleotide supply that is essential for viral replication. Thus, the antiviral effect of DHODH inhibitors (some of which are in preclinical/clinical development) is well known and has been studied for several examples of human pathogenic viruses [5,6,7,8,9,10,11,12,13,14,15]. Notably, DHODH inhibitors have also been characterized for their in vitro activity against coronaviruses [6,15]. Human infection with the severe acute respiratory syndrome coronavirus type 2 (SARS-CoV-2) causes coronavirus disease 2019 (COVID-19), which was declared as a pandemic by the World Health Organization on 11 March 2020. Besides the ongoing development of drug candidates directed against viral targets, such as the authorized drug remdesivir, the concept of developing novel host-cell-directed antivirals (HDAs), which potentially exert broad-spectrum antiviral activity independent of viral mutations appears particularly promising [16,17,18,19,20,21,22,23,24,25,26,27,28,29]. Such purposeful drug profiles, which for some HDAs span over other anti-infective, antitumor and immunoregulatory activities, are considered to be particularly powerful to combat emerging viral diseases such as COVID-19. In the present study, we demonstrate that IMU-838, an inhibitor of the human metabolically and immunologically relevant enzyme DHODH, is efficacious in cultured-cell-based SARS-CoV-2 models. In addition, we show that the active moiety of IMU-838, vidofludimus, is also active against additional human pathogenic viruses, such as human cytomegalovirus (HCMV), human immunodeficiency virus type 1 (HIV-1) and hepatitis C virus (HCV), thus strongly suggesting a broad-spectrum antiviral activity of this class of drugs. Thus, the mode of broad activity is likely linked to this mechanism of nucleotide starving of virus-infected host cells. IMU-838 is currently being investigated in a phase 2 study in COVID-19 patients (NCT04379271). In this report, the characteristics of antiviral in vitro properties are described, and the putative relevance of findings for the development of a SARS-CoV-2-directed therapy option is discussed.

## 2. Materials and Methods

### 2.1. Compounds

In vitro inhibition of human DHODH (hDHODH) was measured using an N-terminally truncated recombinant hDHODH enzyme as described [30,31]. The DHODH inhibitors IMU-838, IMU-CO2, IMU-CO3 and IMU-CO4 were provided by Immunic Therapeutics (Gräfelfing, Germany). IC_50_ values on hDHODH were determined as 160 nM for IMU-838, 240 nM for IMU-CO2, 41 nM for IMU-CO3 and 31 nM for IMU-CO4. Teriflunomide was purchased from Selleck Chemicals (Munich, Germany). Chloroquine (CQ, Sigma-Aldrich, St. Louis, MO, USA) and remdesivir (RDV, Gilead Sciences, Inc., Foster City, CA, USA) were used as reference compounds for anti-SARS-CoV-2 in vitro activity.

### 2.2. SARS-CoV-2-Specific Replication Assay

All infection experiments were performed under biosafety level (BSL)-3 conditions (laboratory M.M.: Regierung von Unterfranken, Würzburg, Az 821-8760.00-23/9, permit BS2344/2020-N; laboratory Z.R.: Regierungspräsidium Tübingen, permit UNI.FRK.05.16/05). Vero B4 and 76 cells (DSMZ ACC33 and ATCC CRL-1587™) were cultivated at 37 °C, 5% CO_2_ and 80% humidity using Dulbecco’s modified Eagle medium (DMEM, 11960044, Thermo Fisher Scientific, Waltham, MA, USA). Cell culture medium was supplemented with 2 mM GlutaMAX^TM^ (35050038, Thermo Fisher Scientific), 10 µg/mL gentamycin (22,185.03, SERVA, Heidelberg, Germany) and 10% fetal bovine serum (FBS, F7524, Sigma-Aldrich, St. Louis, MO, USA). SARS-CoV-2 (MUC-IMB-1/2020, passage ER-P2-2, Bundeswehr Institute of Microbiology, Munich, Germany; or other clinically relevant isolates used in individual experiments as indicated) was propagated on Vero E6 cells (ATCC^®^ CRL-1586) in DMEM with 2% fetal calf serum (FCS). For virus stocks, the cells were infected with a multiplicity of infection (MOI) of 0.001, supernatants were harvested after 50 h and aliquots were stored at −80 °C. These cells were also used for the viral plaque or yield reduction assays. In addition, CaCo-2 cells (ATCC^®^ HTB-37™) were cultivated in DMEM (Thermo Fisher Scientific) supplemented with 10% FCS (Thermo Fisher Scientific) at 37 °C and 5% CO_2_. For infection experiments, Vero cells were seeded in 96-well plates and inoculated with SARS-CoV-2 at different MOIs typically in the range of 0.003 to 0.0002 depending on the intended duration of infection. Infection volumes of 200 µL were used, and viral inoculums remained on the cells. Supernatants and cells were harvested at several time points as indicated. For further downstream analysis, cells were washed with phosphate-buffered saline (PBS) and fixed with 10% formalin (Sigma-Aldrich, St. Louis, MO, USA) at room temperature overnight. Viral supernatants were inactivated for 10 min at 95 °C in sealed plates.

### 2.3. RT-qPCR for the Detection of Extracellular SARS-CoV-2

Prior to RT-qPCR, inactivated viral supernatants were digested with proteinase K (final concentration of 0.136 mg/mL, Sigma-Aldrich, St. Louis, MO, USA) for 1 h at 56 °C followed by 5 min heat inactivation at 95 °C and a dilution of 1:10 in H_2_O. For further analysis, volumes of 5 µL of the digested supernatants were used, and the RT-qPCR was performed according to AgPath-ID™ One-Step RT-PCR (Thermo Fisher Scientific, AM1005). Primer sequences were taken from Corman et al. [32] (RdRp_SARSr-F and RdRp_SARSr-R). The probe (caggtggaacctcatcaggagatgc) was 5′ labeled with 6-FAM (6-carboxyfluorescein) and 3′ with BHQ-1 (Black Hole Quencher 1). All oligonucleotides were purchased from Biomers.net (Ulm, Germany).

### 2.4. In-Cell Immunostaining for the Detection of Intracellular SARS-CoV-2

In-cell immunostaining for quantitation of SARS-CoV-2 replication was performed similar to previously described experimental approaches [33] with the additional implementation of fluorescence labels allowing the parallel detection of multiple antigens and microscopic imaging of infected cells. For quantitation and visualization of intracellular viral antigens or RNA, formalin-fixed cells were washed with PBS, permeabilized with 0.2% Triton X-100 (Carl Roth, Karlsruhe, Germany) in PBS and blocked with blocking buffer (1% bovine serum albumin, BSA, in PBS, Sigma-Aldrich, St. Louis, MO, USA). Cells were sequentially incubated with primary and secondary antibodies diluted in blocking buffer, with two PBS washing steps after each antibody incubation. Different combinations of primary and secondary antibodies were evaluated depending on the desired readout system. For the detection of SARS-CoV-2 antigens by a convalescent antiserum, an HRP-labeled anti-human secondary antibody and TMB substrate (BioLegend, San Diego, CA, USA) for colorimetric readout were used (in-cell ELISA). A new mouse monoclonal antibody against the viral spike protein (mAb-S TRES-6.18) was produced on the basis of a standard mouse immunization scheme [34]. The mouse was primed with a plasmid encoding the wild-type SARS-CoV-2 spike protein (SARS-CoV-2 Wuhan, position 21,580–25,400, GenBank NC_045512) and boosted twice with the Spike protein of SARS-CoV-2 stabilized in a pre-fusion conformation (details about antibody characteristics will be described elsewhere). For the specific quantitation of the viral spike protein (in-cell indirect immunofluorescence [IF] protein detection) or double-stranded RNA (in-cell IF dsRNA detection), mouse mAb-S IgG isotype 2c or the J2 IgG2a mouse monoclonal antibody for dsRNA, which is specific for RNA helices of at least 40 bp and inert toward other nucleic acid species in uninfected cells, (SCIONS, Szirák, Hungary) [35] was used as a primary antibody with an Alexa 488-conjugated secondary antibody for detection. For simultaneous staining of the spike protein and dsRNA, an IgG2a-specific Alexa 555-labeled antibody (Invitrogen) and an IgG 2c-specific Alexa 488-conjugated antibody (Jackson ImmunoResearch Laboratories, Inc.) were used in combination. In some experiments, an optimized spike and dsRNA detection was achieved by consecutive incubation steps, including an intermediate antibody stripping step by incubation with 62.5 mM Tris pH 6.8 (Carl Roth, Karlsruhe, Germany), 100 mM β-mecaptoethanol (Promega, Madison, WI, USA) and 2% SDS (SERVA, Heidelberg, Germany) at 37 °C for 2 h followed by several washing steps with PBS [36]. The effectiveness of primary antibody stripping was monitored by a control reprobing stain exclusively with a secondary antibody. Stained plates were quantitatively evaluated in a Victor X4 multilabel reader (Perkin Elmer, Waltham, MA, USA) and used for imaging by an ImmunoSpot^®^ S6 ULTIMATE UV Image Analyzer (Cellular Technology Limited/CTL, Cleveland, OH, USA). Parallel to antibody staining, one of the two fluorescent DNA dyes SYTOX Blue or Hoechst 33342 (both Thermo Fisher Scientific ) was used as an internal control for estimating cell counts in Victor X4 or the Immunspot reader measurements, respectively.

### 2.5. Western Blot Analysis

Western blot analysis (Wb) was performed by standard procedures as described previously [37,38,39]. Human convalescent antiserum (1:3000) in combination with an horseradish peroxidase (HRP)-labeled secondary antibody was used for detection.

### 2.6. Viral Plaque and Yield Reduction Assays

To assess the viral activity of the compounds and the combination of IMU-838 and remdesivir (RDV, MedChem Express, Monmouth Junction, NJ, USA) against SARS-CoV-2, a viral yield reduction (VYR) assay was performed. Hereby, the supernatants from each compound concentration or combination was collected at 3 days post-infection (p.i., 3 wells pooled). Virus titers were quantitated using a standard endpoint dilution assay and titer calculations using the Reed–Muench equation [40] after 5 days. The concentration of compound required to reduce virus yield by 1 log_10_ was calculated by regression analysis. To assess the inhibitory activity of IMU−838 against SARS-CoV-2 in infected CaCo-2 cells, which were supplied with fresh medium (DMEM with 2% FCS and 20 mM HEPES) containing 7.5, 15, 30 or 60 µM IMU-838 at 1.5 h p.i., the infectious supernatants were harvested 72 h p.i.. To determine viral loads, a plaque assay was performed by serial dilutions of the supernatant in PBS and incubation of the dilutions on Vero E6 monolayers for 1.5 h at room temperature. The infection inoculums were removed, and the cells were overlaid with medium containing a 0.6% oxoid agar solution and 1% FCS. After incubation for 72 h at 37 °C, cells were fixed with formaldehyde (3.7% in PBS) and stained with 0.1% crystal violet (Sigma-Aldrich).

### 2.7. Neutral Red Assay (NRA)

Cytotoxicity of the analyzed compounds was determined by the approved dye uptake assay using Neutral Red (NRA). Vero cells were seeded in 96-well plates one day prior to testing, cultivated overnight until cells were ~80% confluent and then incubated with test compounds for 3 or 5 days. The assay was performed as described previously [41] using 40 µg/mL Neutral Red (Sigma Aldrich, St. Louis, MO, USA). The amount of incorporated Neutral Red was quantitated in a microplate reader by fluorescence measurement using 560/630 nm for excitation/emission, respectively.

### 2.8. Methods for Comparative Antiviral Analysis of Additional Human Pathogenic Viruses

Replication of HCMV was performed by infecting primary human foreskin fibroblasts (HFFs) with the HCMV AD169-GFP reporter virus using the cell-associated fluorescence at 7 days p.i. as a readout for viral replication or by standard plaque reduction assays according to previously established protocols [42,43,44]. HCV replication was performed in the Huh7 human hepatoma cell line containing a HCV subgenomic replicon of genotype 1b with a stable luciferase (Luc) reporter and three cell-culture-adaptive mutations (Luc-ubi-neo ET, under license from Ralf Bartenschlager and Apath, LLC) [45]. Viral replication was detected by measurement of replicon-derived luciferase activity 24 h p.i. as readout. For HIV-1 replication was performed in phytohemagglutinin (PHA)-stimulated human peripheral blood mononuclear cells (PBMCs) from HIV and hepatitis B virus (HBV) seronegative donors, infected with a low-passage stock of the clinical virus isolate HIV-1_91US005_ (CCR5-tropic, subtype B, National Institutes of Health (NIH) acquired immunodeficiency syndrome (AIDS) Reagent Program, Division of AIDS, NIAID, NIH courtesy of Dr. Beatrice Hahn and the DAIDS). After 7 days, supernatants were collected for analysis of cell culture supernatant-associated viral reverse transcriptase (RT) activity according to previously described protocols [46]. Additionally, viral p24 antigen was measured using an ELISA kit (XpressBio Life Science Products, Frederick, ML, USA). For further details on cell culture conditions, readout systems and evaluation of viral replication see the Supplementary Materials section.

### 2.9. Statistical Analysis

Data for the VYR assay with CaCo-2 cells were visualized and statistically evaluated with GraphPad Prism 7 (GraphPad Software, San Diego, CA, USA). Viral titers of the growth kinetics were displayed as log-transformed values on a linear scale (mean with standard deviation). Statistics were computed by a one-way ANOVA using the Tukey’s multiple comparison test. To determine the reduction to DMSO, the means were subtracted from the DMSO control and expressed as exponents for the power of 10.

## 3. Results

### 3.1. Establishment of a Cell-Culture-Based SARS-CoV-2-Specific Replication Assay

**Figure 1:**
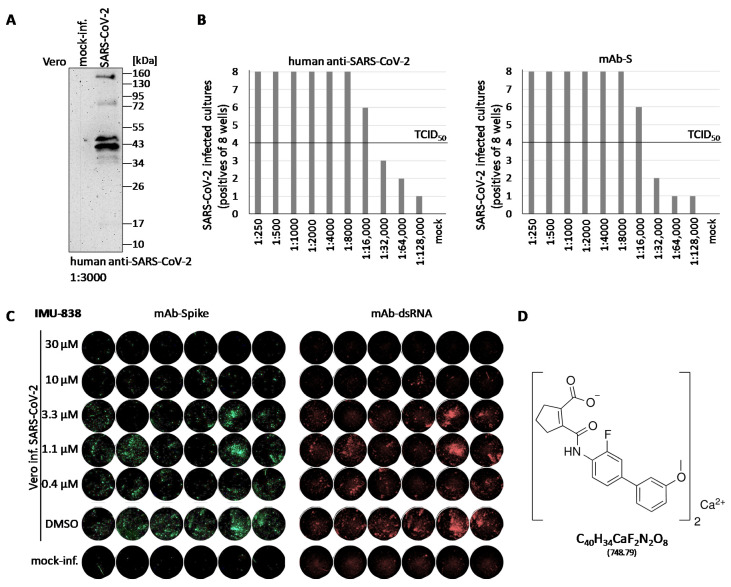
Use of a clinical isolate of severe acute respiratory syndrome coronavirus type 2 (SARS-CoV-2) for cell-culture-based infection assays. (**A**) Demonstration of productive viral replication. Vero cells were seeded in T25 flasks (2.5 × 10^6^ cells), used for infection with SARS-CoV-2 at a multiplicity of infection (MOI) of approx. 1 and harvested at 2 days post-infection (p.i.). Total lysates were subjected to standard SDS-PAGE/Western blot analysis and immunostained using a human convalescent antiserum at a dilution of 1:3000. (**B**) Titration of infectious viral stocks. SARS-CoV-2 infection was performed on Vero cells cultured in a 96-well plate applying serial dilutions as indicated. Cells were harvested at 2 days p.i. and virus-specific immunostaining was performed by in-cell ELISA using human anti-SARS-CoV-2 or by in-cell IF staining using an monoclonal antibody against the viral spike protein (mAb-S), as indicated, to determine numbers of infected cultures for subsequent calculation of tissue culture infectious doses (TCID_50_/mL). (**C**) In-cell IF assay, using mAb-S (left panel) and mAb-dsRNA (right panel), showing the concentration-dependent IMU-838-mediated inhibition of viral spike protein or RNA production, respectively. Vero cells were seeded in 96-well plates one day before infection (3 × 10^4^ cells/well) with SARS-CoV-2 at an MOI of 0.003 and treated with the concentrations of 30, 10, 3.3, 1.1 and 0.4 µM of IMU-838. At 30 h p.i., cells were fixed and used for the IF antibody staining. DMSO, viral replication control with solvent only; mock-inf., uninfected cells used as a background negative control. (**D**) Structure of IMU-838, which is the calcium salt of vidofludimus.

In the first step, a suitable analysis system was established for the investigation of putative SARS-CoV-2-inhibitory drugs. A clinical isolate of SARS-CoV-2 (MUC-IMB-1/2020) was further amplified to high-titer virus stocks and then employed for infection assays in Vero cells. We used two sublineages of Vero, namely B4 and 76, and found that these were comparably susceptible to SARS-CoV-2 in vitro infection under the chosen conditions. Productive infection was monitored by Western blot analysis using a human anti-SARS-CoV-2 convalescent serum (Figure 1A), and viral stocks were quantitated by titration of infectious units using human anti-SARS-CoV-2 convalescent antiserum in an in-house developed in-cell ELISA or a newly produced monoclonal antibody against the viral spike protein (mAb-S) for in-cell indirect immunofluorescence (IF) staining (Figure 1B). We directly included IMU-838 in this assay establishment, as it represented the most promising multi-potent developmental drug of the project. The first indication for a SARS-CoV-2-directed activity of IMU-838 was obtained by assessing its inhibitory potential by in-cell IF staining using mAb-S (Figure 1C, left panel) and mAb-dsRNA (right panel). Both stainings demonstrated the concentration-dependent IMU-838-mediated inhibition of the respective viral expression products. On this basis, a test system for SARS-CoV-2 infection of Vero cells was obtained in a 96-well plate setting under conditions described in the Material and Methods section.

**Figure 2:**
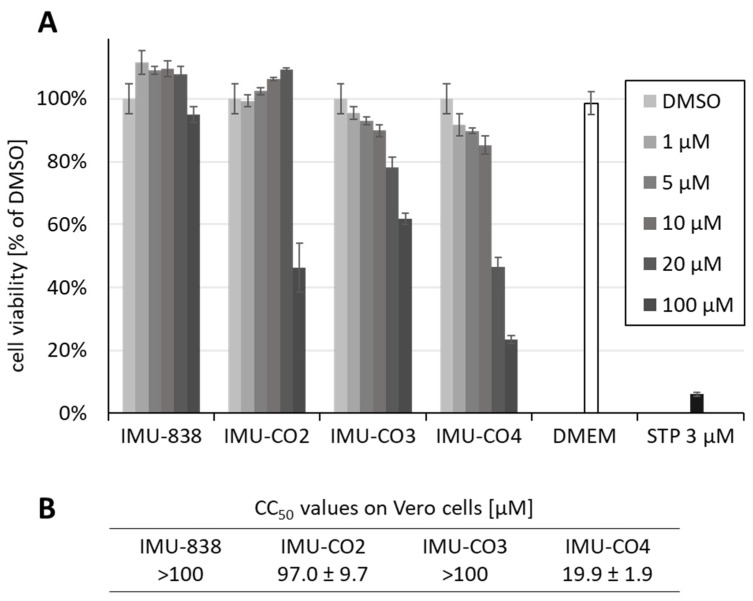
Determination of cytotoxicity levels of IMU-838 and other DHODH inhibitor back-up compounds using the Neutral Red assay (NRA) on Vero cells. (**A**) Subconfluent layers of Vero cells were cultivated in 96-well plates, incubated with the indicated concentrations of IMU-838, IMU-CO2-4, the cytotoxic reference drug staurosporine (STP) or solvent DMSO for 3 days and subjected to NRA measurement in triplicates (Dulbecco’s modified Eagle medium (DMEM), additional medium control). Mean values ± SD are given. (**B**) Levels of cytotoxicity determined for IMU-838 and IMU-CO2-4 on confluent layers of Vero cells under the same conditions as in panel A. Mean half-maximal cytotoxic concentrations (CC_50_ values) are given.

In conjunction with the antiviral tests, the levels of putative drug cytotoxicity were assessed using the NRA. Uninfected Vero cells were treated with compounds at a concentration range between 1 and 100 µM. The prototype drug IMU-838 showed minimal cytotoxicity on subconfluent layers of Vero cells (CC_50_ >100 µM, 95% viability at 100 µM; Figure 2A). Similarly, the cytotoxicity values of other pharmacological candidates of DHODH inhibitors remained at a tolerable level, i.e., 97.0, >100 and 19.9 µM, respectively (Figure 2B; IMU-CO2-4 compounds were derived from the Immunics small-molecule screening library, see Section 2.1). Based on this cytotoxicity profile in combination with particularly low IC_50_ values on hDHODH, the two compounds IMU-CO3-4 were further analyzed concerning anti-SARS-CoV-2 activity (see Section 3.3). Drug cytotoxicity was further determined by confirmatory NRA analyses, in which 3 day and 5 day treatments were compared, and data referring to the two time points did not indicate substantial differences (data not shown). This result was also confirmed by regular microscopic inspection of drug-treated cell layers, performed with both mock-infected and SARS-CoV-2-infected cells, thus underlining the lack of detectable cytotoxicity within the relevant range of concentrations.

### 3.2. Assessment of the Anti-SARS-CoV-2 Activity of IMU-838

**Figure 3:**
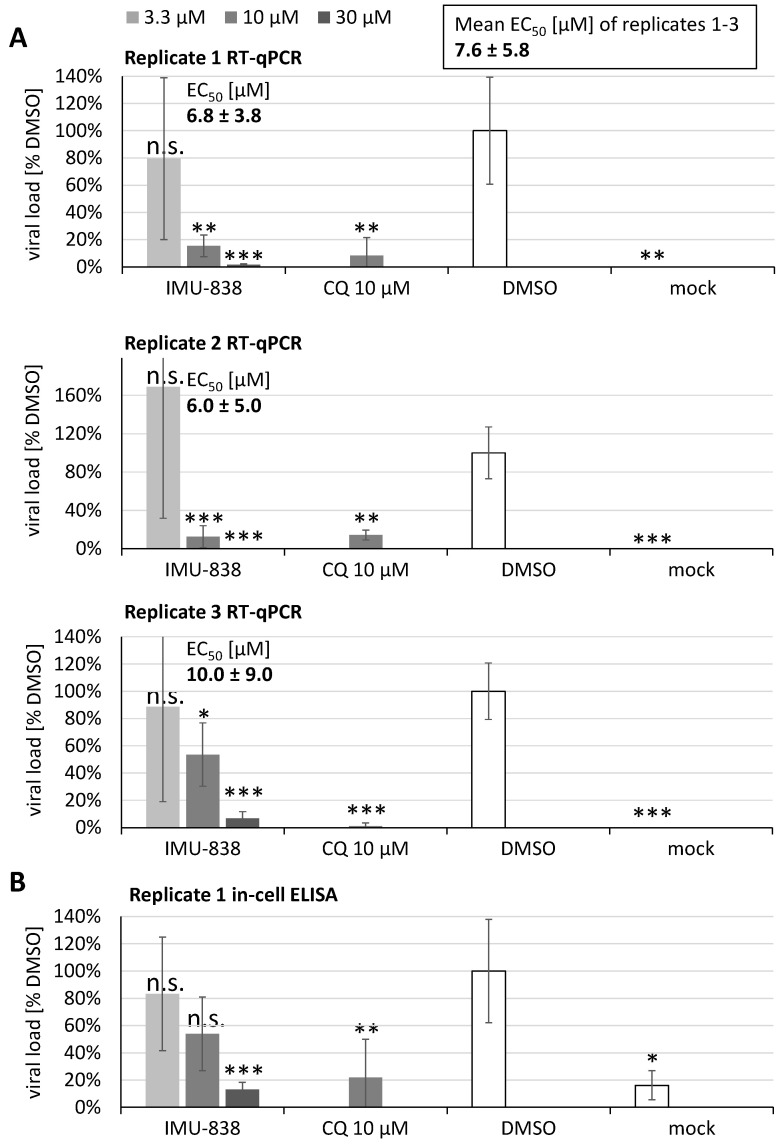
Antiviral activity of IMU-838 determined by RT-qPCR using infectious supernatants of SARS-CoV-2-infected Vero cells, and subsequently by in-cell ELISA using the remaining cell layers. Cells were seeded in 96-well plates one day before infection (4 × 10^4^ cells/well), then used for infection with SARS-CoV-2 at a multiplicity of infection (MOI) of 0.0002 and treatment with the concentrations of 30, 10 and 3.3 µM of IMU-838 or 10 µM of chloroquine (CQ) as a control and further cultivated for 2–3 days. Supernatants were harvested, subjected to proteinase K digest for the isolation of viral RNA and used for the determination of extracellular viral load by RT-qPCR. The mean half-maximal effective concentration (EC_50_) value of the three replicates is given above the panels. (**A**) Replicate 1: SARS-CoV-2 infection/drug treatment of Vero B4 cells measured 2 days post infection (p.i.); replicate 2: SARS-CoV-2 infection/drug treatment of Vero B4 cells measured 3 days p.i.; replicate 3: SARS-CoV-2 infection/drug treatment of Vero 76 cells measured 3 days p.i. The DMSO solvent control was set at 100%. (**B**) The remaining fixed cell layers of replicate 1 in panel A were additionally subjected to the in-cell ELISA using a human anti-SARS-CoV-2 convalescent serum for the confirmation of drug-mediated inhibition of intracellular viral load. Statistical significance was calculated by unpaired Student’s *t*-test by comparing each treatment with DMSO. * *p* <0.05; ** *p* <0.01; *** *p* <0.001; n.s., not significant.

The antiviral activity of IMU-838 was determined by RT-qPCR using infectious supernatants of SARS-CoV-2-infected Vero cells. Cells were used for infection with SARS-CoV-2 at an MOI of 0.0002 for 2–3 days, before supernatants were collected and subjected to the determination of extracellular viral load by RT-qPCR. The inhibitor chloroquine (CQ), solvent control DMSO and a mock-infected control verified the reliability of the assay conditions (Figure 3). In all cases of the slightly modified settings in replicates 1–3, the EC_50_ values of IMU-838 remained in the low micromolar range (6.0 ± 5.0 µM to 10.0 ± 9.0 µM), so that a mean of 7.6 ± 5.8 µM was calculated (Figure 3A). This result was further illustrated by the use of the remaining cell layers for assessing the drug-mediated inhibition of intracellular viral load by an immunostaining of cell layers in the in-cell ELISA (Figure 3B). This finding was supported by the in-cell IF data described above, indicating the IMU-838-mediated inhibition of viral spike protein and RNA production, with a concentration-dependent reduction of both signals, as compared to the DMSO infection-positive and mock-infected negative controls (Figure 1C). These data indicated a pronounced in vitro anti-SARS-CoV-2 activity of the developmental drug IMU-838.

**Figure 4:**
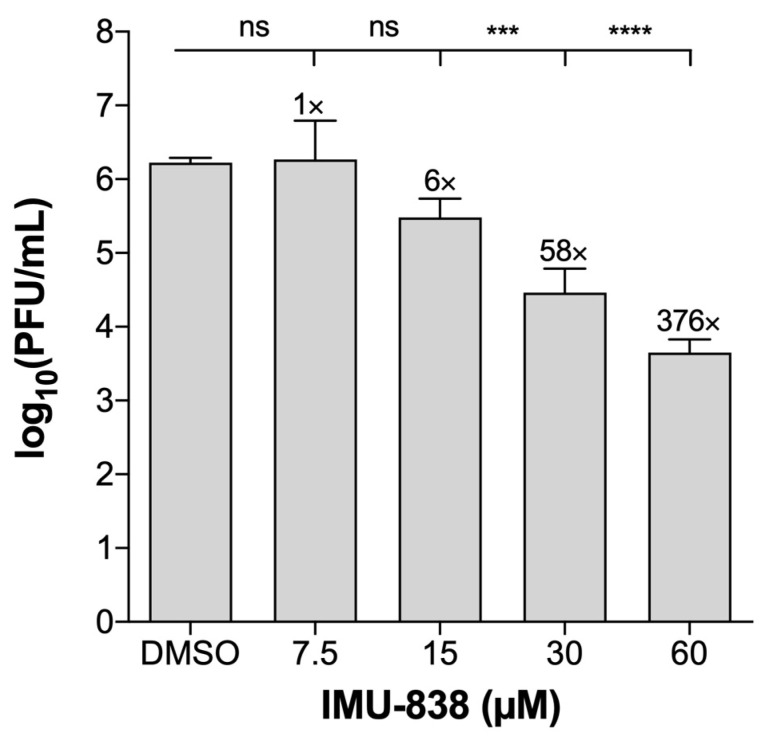
Determination of anti-SARS-CoV-2 activity of IMU-838 in CaCo-2 cells. CaCo-2 cells were infected with SARS-CoV-2 at an MOI of 0.001 for 1.5 h at room temperature. Thereafter, fresh medium containing DMSO or IMU-838 (7.5, 15, 30 or 60 µM) was added. At 72 h p.i., supernatants were collected, and serial dilutions of the supernatants were incubated on a monolayer of Vero E6 cells. After 1.5 h, medium was removed, and cells were incubated for 72 h with fresh medium at 37 °C. After this incubation, cells were fixed and stained with crystal violet, and plaque formation was determined. The test was performed in triplicate (*n* = 3), and absolute values of plaque-forming units (PFU) are given (mean ± SD). Statistical significance is indicated according to one-way ANOVA followed by Tukey’s multiple comparison test: *** *p* <0.001; **** *p* <0.0001; n.s., not significant.

The inhibitory efficacy of IMU-838 on SARS-CoV-2-infected cells was further validated using variable read-out methods in several cell lines and lineages. SARS-CoV-2-infected, drug-treated CaCo-2 cells were used for the transfer of supernatants to monolayers of Vero E6 cells for the quantitation of plaque formation, and in this approach, a ~1.8 log unit reduction (58-fold reduction) of viral plaques was observed for the treatment setting of 30 µM IMU-838 (Figure 4). Moreover, performing a VYR assay with Vero 76 cells (Table 1), a virus-specific EC_90_ of 6.2 ± 1.9 µM was measured, with no cytotoxicity observed with drug concentrations up to 100 µM at 3 days p.i.. Table 1 gives an overview of further collected results.

### 3.3. Additional Assessment of the Anti-SARS-CoV-2 Activity of DHODH Inhibitor Back-up Compounds

**Figure 5:**
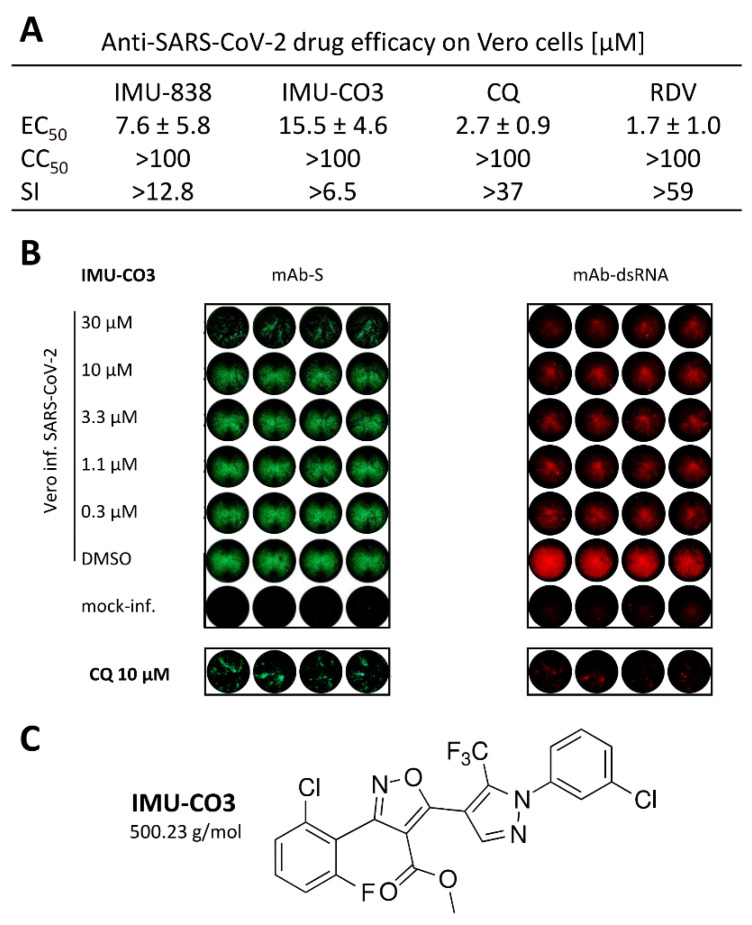
Determination of the anti-SARS-CoV-2 activity of DHODH inhibitor back-up compound, IMU-CO3. As based on the experimentation performed with IMU-838 shown in Figure 2, Figure 3 and Figure 4, a similar in vitro characterization of anti-SARS-CoV-2 activity was performed with IMU-CO3. (**A**) RT-qPCR using infectious supernatants of SARS-CoV-2-infected Vero cells was used to assess the antiviral efficacy in quantitative terms. EC_50_ values ± SD are given and set in relation to CC_50_ and SI values. Values for reference compounds chloroquine (CQ) and remdesivir (RDV) were determined using the same test system. (**B**) In-cell IF stainings using mAb-S and mAb-dsRNA were performed to demonstrate the intracellular antiviral efficacy on the levels of inhibition of viral spike protein production and genomic viral RNA, respectively. Note the double-staining of drug-treated, infected Vero cell wells by the use of isotype-specific secondary fluorescence-labeled antibodies. Control staining is shown for the reference compound CQ. **(C**) Structure of the DHODH inhibitor IMU-CO3.

Further developmental or experimental drug candidates targeting hDHODH were characterized in the SARS-CoV-2 assay system under identical conditions (Figure 5A–C). IMU-CO3 was chosen as a representative example from the Immunic small-molecule screening library with proven DHODH inhibition (see Section 2.1) and structurally unrelated toward IMU-838 (Figure 5C). The determination of antiviral efficacy by RT-qPCR showed a promising antiviral profile with an EC_50_ value of 15.5 ± 4.6 µM (IMU-CO3; Figure 5A). The reference drugs chloroquine (CQ) and remdesivir (RDV) revealed EC_50_ values of 2.7 ± 0.9 µM and 1.7 ± 1.0 µM (RT-qPCR), respectively, which are consistent with previous reports on their anti-SARS-CoV-2 activity in Vero cells (Figure 5A, Figure S1) [47,48,49,50,51]. The IF analysis performed in parallel illustrated this finding by confirming the intracellular antiviral potency of the drug (Figure 5B), as exemplified on the levels of inhibited viral protein (mAb-S) and viral double-strand RNA (mAb-dsRNA). These two approaches of in-cell IF immunodetection, together with the RT-qPCR data, clearly indicated for IMU-838 and IMU-CO3 a drug-mediated reduction of three virus-specific signal levels, i.e., viral protein production, dsRNA synthesis and infectious virus release (Figure 1, Figure 2 and Figure 3 and 5, Table 1). Furthermore, the inhibitory efficacy of additional back-up compounds on SARS-CoV-2-infected cells was analyzed in parallel, and the data confirmed our findings of anti-SARS-CoV-2 activity exerted by DHODH inhibitors, with IMU-CO4 representing another candidate with strong antiviral in vitro efficacy (EC_50_ 7.5 ± 0.7 µM; data not shown). In essence, the findings of this study demonstrate the potency of this type of inhibitor as potential anti-SARS-CoV-2 agents, with the main focus on developmental drug IMU-838, which is presently under clinical investigation also including COVID-19 patients (NCT04379271).

### 3.4. Combinatorial and Broad-Spectrum Aspects of IMU-838 Antiviral Activity

**Figure 6:**
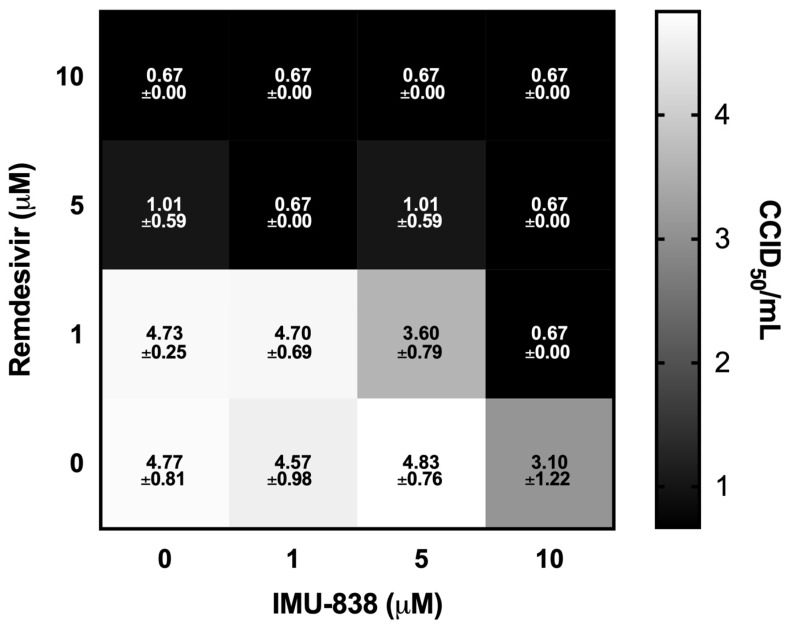
Combinatorial antiviral in vitro effect obtained through IMU-838−RDV treatment of SARS-CoV-2-infected Vero cells. A viral yield reduction assay using a standard endpoint dilution assay and calculating the virus titer units at 50% cell culture infectious doses (CCID_50_) per mL. Vero 76 cells were cultured for 3 days with DMSO control, 1, 5 or 10 µM IMU-838 or RDV either alone or in combination. Data represent mean values ± SD of determinations in triplicate (*n* = 3). All squares showing a CCID_50_/mL of 0.67 were below the detection limit of 0.67.

Of note, a recent publication suggested an additive or even synergistic effect for combination treatments of DHODH inhibitors with direct-acting antivirals (DAAs; [15]). In this regard, an initial analysis was performed in the context of this study for assessing the efficacy of IMU-838 in combination with RDV against SARS-CoV-2. RDV was chosen as the DAA in a viral yield reduction assay. While IMU-838 alone showed ~1.7-fold log unit reduction at 10 µM and RDV alone a ~3.8 and >4.0 fold log unit reduction with 5 and 10 µM, respectively, the combination effect of IMU-838−RDV was found to have an enhanced manner of antiviral activity based on the combinatorial effect of the two mechanistically different drugs. Notably, at 1 µM of RDV combined with 10 µM of IMU-838, effected an almost complete reduction in viral yield (>4.0-fold log units), and a similar efficacy was obtained for 5 µM of RDV combined with 1 µM of IMU-838 (Figure 6). Thus, the finding strongly suggested highly promising potential of this drug combination in vitro.

An increasing amount of in vitro evidence was published indicating that DHODH inhibitors might be potent broad-spectrum antivirals. This broad activity would make DHODH inhibitors useful to control the mortality burden of newly emerging or re-emerging pandemics. To better assess a potential broad-spectrum antiviral activity of IMU-838 or its active moiety, vidofludimus, multiple in vitro assays with various viruses were performed (Table 2). Vidofludimus exhibited an EC_50_ value of 7.4 µM against HCMV in HFFs in the absence of detectable cytotoxicity. Likewise, the activity of vidofludimus was tested against HCV in Huh7 cells, with similarly promising results, indicating an EC_50_ value of 5.9 µM and only a reduction of 34% in cell viability with the highest concentration of vidofludimus (30 µM). Finally, the efficacy of vidofludimus was analyzed for HIV-1 in human PBMCs, resulting in an EC_50_ of 2.1 µM with a reverse transcriptase endpoint assay and 1.3 µM for the p24 ELISA endpoint detection method. No CC_50_ could be determined (>100 µM) on the basis of low cytotoxicity in the range of concentrations relevant for antiviral activity, but only at the highest concentration (100 µM), a reduction of 46.8% in cell viability was observed.

## 4. Discussion

This study provides first experimental evidence that the developmental DHODH inhibitor IMU-838 shows antiviral in vitro efficacy, particular against SARS-CoV-2. Moreover, the data presented here, together with the recently published antiviral activity of IMU-838 against arenaviruses [52], implicate that the active moiety of IMU-838, vidofludimus, possesses broad-spectrum antiviral activity. Given the current COVID-19 pandemic, it is important to emphasize that the anti-SARS-CoV-2 in vitro activity of IMU-838 is detectable at concentrations that are known to be exceeded by drug levels in our clinical trials [1,2,53]. As far as the mechanistic aspect of this novel HDA candidate is concerned, virus-infected cells are highly metabolically active and, thus, show dependence toward intracellular DHODH activity to sustain their high metabolic turnover [5]. In these cells, the extraordinary demand of nucleotides cannot be sufficiently supported by metabolic recycling, but *de novo* pyrimidine synthesis needs to be activated. Especially, sufficient nucleotide supply by the host cell, representing a critical requirement for the replicative steps of viruses including the syntheses of viral genomes, transcripts and proteins, is considered a promising mode of antiviral action through the blocking of DHODH activity by pharmacological DHODH inhibitors (Figure S2).

In addition to the in vitro observations, Xiong et al. [15] have shown that the DAA oseltamivir was primarily effective in the early phase of influenza A virus infection in an influenza mouse model (i.e., within 48 h of symptom onset), while DHODH inhibitors may also be effective when treatment was started in middle-to-late phases of influenza disease [15]. This could be a beneficial feature of DHODH inhibition in viral infection, since patients may rather initiate antiviral treatment in a later phase, when the symptoms have manifested, than at early time points. For the case of severe influenza A virus infections in mice, a recent report indicated an additive or even synergistic effect for combination treatments using DHODH inhibitors with influenza DAAs [15]. In this regard, we performed an initial analysis for assessing the efficacy of IMU-838 in combination with RDV against SARS-CoV-2. RDV represents an authorized COVID-19 drug for emergency use and was chosen as the DAA in a viral yield reduction assay. The examination of IMU-838−RDV combination effects indicated a promising profile in this first-time approach in vitro, thus pointing to the option of a combination treatment for severe SARS-CoV-2 infections in vivo.

Besides the depletion of nucleotide pools for viral genome synthesis, blocking the pyrimidine synthesis was also demonstrated to activate the innate immune response by upregulation of interferon-inducible antiviral genes [8,9,54,55]. This indirect antiviral effect involving the interferon regulatory factor 1 (IRF1) transcription factor and induction of endogenous interferons to induce a broad host-mediated antiviral cellular state might additionally contribute to the antiviral activity of IMU-838 and other DHODH inhibitors [53,56]. Concerning clinical aspects, severe cases of COVID-19 have been linked to hyperactivation of the immune system with excessive cytokine production for progression to the acute respiratory distress syndrome and multiorgan failure. Since IMU-838 was developed to pharmacologically interfere with metabolically hyperactivated immune cells to treat autoimmune diseases, it might also be applied to reduce the cytokine storm induced by viral infections. This notion is supported by recent reports demonstrating that IMU-838 reduces T lymphocyte proliferation, cytokine production and organ infiltration by leukocytes in various in vivo and in vitro models for autoimmunity [53,56]. Additional immune modulatory effects of vidofludimus including decreased inflammation in the lung were demonstrated in a mouse model of systemic lupus erythematosus [57], findings that are consistent with previous reports on other DHODH inhibitors [58,59]. Thus, it could be speculated that IMU-838 also reduces inflammation in lung tissue in the context of SARS-CoV-2 infections in a similar way. In this context, it should be emphasized that elevated levels of myeloperoxidase (MPO)–DNA complexes are associated with the need for ventilation in COVID-19 patients [60]. In a murine 2,4,6-trinitrobenzene sulfonic acid (TNBS)-induced colitis model, vidofludimus treatment reduced the levels of MPO in colonic tissue, thus implicating similar effects on immune cell hyperactivation in other relevant clinical situations [57].

Finally, it should be stressed that the anti-SARS-CoV-2 data presented here were obtained in three different laboratories, indicating that these data are robust in terms of being produced using different cell systems, readouts and facilities. Based on the mode of action of IMU-838 as an HDA-type DHODH inhibitor, it is expected that even facing substantial rates of virus mutations, IMU-838 would still retain its antiviral activity. In addition, we also show that our back-up DHODH inhibitor compounds are potent against SARS-CoV-2, although their efficacy seems to be lower. Although all compounds represent potent inhibitors of DHODH in vitro in a cell free assay, differences in the cellular or specifically in the mitochondrial uptake most likely account for these differences in antiviral activity.

To further emphasize the medical applicability of this class of drugs, the use of IMU-838 is in ongoing phase 2 clinical development, with more than 650 persons treated so far, and appears highly promising. The adverse event profile is on a similarly low level when compared to the placebo group [2]. This is considered as a relevant achievement, since existing DHODH inhibitors, such as brequinar and leflunomide/teriflunomide, previously showed rather high levels of cytotoxicity and/or an unfavorable profile of pharmacokinetics. In case of IMU-838, our clinical data strongly suggested therapeutic efficacy in a phase 2 clinical trial for multiple sclerosis (EMPhASIS, NCT03846219). At present, there are also three phase 2 clinical trials ongoing with applications in ulcerative colitis (CALDOSE-1, NCT03341962), primary sclerosing cholangitis (an investigator-sponsored trial, NCT03722576) and COVID-19 (CALVID-1, NCT04379271). Overall, IMU-838 is a potent DHODH inhibitor nominated as a highly interesting developmental candidate for the treatment of specific diseases including COVID-19, as particularly focused by the CALVID-1 trial (NCT04379271) intended for the clinical benefit of COVID-19 patients.

## Figures and Tables

**Table 1:** Overview of IMU-838 efficacy in cultured-cell-based SARS-CoV-2 infection models. ^a^

Cells	SARS-CoV-2 Isolate	MOI	Assay	Cmp	EC_50_ (µM)	CC_50_ (µM)	SI_50_	EC_90_ (µM)	CC_90_ (µM)	SI_90_
**Vero 76** ^b^	USA_WA1/2020	0.002	VYR	IMU-838	-	-	-	6.2 ± 1.9	>100 ^e^	>16.1
				TFNM	-	-	-	14	-	-
**Vero 76** ^c^	MUC-IMB-1	0.0002	RT-qPCR	IMU-838	10.0 ± 9.0	88.1 ± 3.7	>10.0	-	-	-
**Vero B4** ^c^	MUC-IMB-1	0.0002	RT-qPCR	IMU-838	6.8 ± 3.8 ^f^ 6.0 ± 5.0 ^g^	>100	>14.7	-	-	-
**CaCo-2** ^d^	MUC-IMB-1	0.001	VYR	IMU-838	7.5–15	>60 ^e^	>4–8	15–30	>60 ^e^	>2–4

Values are presented as mean ± SD or alternatively as intervals reflecting the variability. ^a^ Experiments were performed in three independent laboratories at coauthor affiliations: ^b^ B.H., J.J.; ^c^ F.H., C.W., S.H., A.S.P., G.D., K.Ü., H.M.J., M.M; ^d^ J.F., Z.R. ^e^ Determined by visual microscopic inspection. ^f^ 2 days, ^g^ 3 days. Abbreviations: VYR, viral yield reduction assay; TFNM, teriflunomide; EC_50_, half-maximal effective concentration; CC_50_, half-maximal cytotoxic concentration; SI_50_, selectivity index CC_50_/EC_50_.

**Table 2:** Broadness of antiviral activity of vidofludimus analyzed for human cytomegalovirus (HCMV), hepatitis C virus (HCV), and human immunodeficiency virus type 1 (HIV-1).

Virus ^a^	Family	Cells ^b^	Assay ^c^	Drug ^d^	EC_50_ (µM)	CC_50_ (µM)	SI
HCMV	*Herpesviridae*	HFF	GFP-based replication assay	Vido	7.4 ± 0.1	>100	>13.5
				GCV	1.69 ± 0.8	>15 ^e^	>8.8
HCV	*Flaviviridae*	Huh7	Luc reporter assay	Vido	5.9 (4.5–7.7)	>30	>5.1
				rIFNα-2b ^f^	0.21 (0.17–0.26)	2	>9.5
HIV-1	*Retroviridae*	Human PBMC	RT assay	Vido	2.1 (1.0–4.4)	>100	>47.5
				AZT	0.38 × 10^−3^ (0.20 × 10^−3^– 0.63 × 10^−3^)	>1	>2655
			p24 ELISA	Vido	1.3 (0.6–2.7)	>100	>76.1
				AZT	0.27 × 10^−3^ (0.10 × 10^−3^– 0.53 × 10^−3^)	>1	>3693

Values are presented as mean ± SD (or alternatively as 95% confidence intervals reflecting the variability). ^a^ Viruses used: HCMV, human cytomegalovirus (M.M.); HCV, hepatitis C virus (R.P.); HIV-1, human immunodeficiency virus type 1 (R.P.). ^b^ Cell types used: HFF, primary human foreskin fibroblasts; Huh7, human hepatoma cell line; PBMC, peripheral blood mononuclear cell. ^c^ Assay systems applied: HCMV green fluorescent protein (GFP)-based replication assay [43]; luciferase (Luc) reporter assay, RT assay and p24 ELISA (see Materials and Methods section). ^d^ Abbreviation of drug names: Vido, vidofludimus (active moiety of IMU-838); GCV, ganciclovir; rIFNα-2b, recombinant interferon α-2b; AZT, azidothymidine/zidovudine (the latter three used as reference controls). ^e^ CC_50_ value of GCV in HFF has been reported previously [28]. ^f^ rIFNα-2b activity is indicated as IU/mL.

## Supplementary Materials

The following are available online at https://www.mdpi.com/1999-4915/12/12/1394/s1, Figure S1: Antiviral activity and cytotoxicity determinations of reference drugs, Figure S2. Schematic depiction of the mode of inhibitory activities of DHODH-directed drugs, Supplementary Methods. Methods for comparative antiviral analysis of human pathogenic viruses.
