# Supplementary material for: IMU-838, a Developmental DHODH Inhibitor in Phase II for Autoimmune Disease, Shows Anti-SARS-CoV-2 and Broad-Spectrum Antiviral Efficacy In Vitro
Source: Viruses. 2020 Dec 5;12(12):1394. doi: 10.3390/v12121394 (PMC7762174; doi:10.3390/v12121394)
Supplement: Supplementary file 1 [file viruses-12-01394-s001.pdf]

Supplementary Material

IMU-838, a developmental DHODH inhibitor in phase II for autoimmune disease, shows anti-SARS-CoV-2 and broad-spectrum antiviral efficacy *in vitro*

Friedrich Hahn [1], Christina Wangen [1], Sigrun Häge [1], Antonia Sophia Peter [1], Gerhard Dobler [2], Brett Hurst [3], Justin Julander [3], Jonas Fuchs [4], Zsolt Ruzsics [4], Klaus Überla [1], Hans-Martin Jäck [5], Roger G. Ptak [6], Andreas Muehler [7], Manfred Gröppel [7], Daniel Vitt [7], Evelyn Peelen [7], Hella Kohlhof [7,&], Manfred Marschall [1,&,*]

[1] Institute for Clinical and Molecular Virology, Friedrich-Alexander University of Erlangen-Nürnberg (FAU), Schlossgarten 4, 91054 Erlangen, Germany (friedrich.hahn@uk-erlangen.de, christina.wangen@uk-erlangen.de, antoniasophia.peter@uk-erlangen.de, klaus.ueberla@fau.de, sigrun.haege@fau.de, manfred.marschall@fau.de);

[2] Institute of Microbiology, Bundeswehr, Neuherbergstraße 11, 80937 München, Germany (gerharddobler@bundeswehr.org);

[3] Institute for Antiviral Research, Utah State University, 5600 Old Main Hill, Logan, UT 84322, USA (brett.hurst@usu.edu, justin.julander@usu.edu);

[4] Institute for Virology, University Medical Center/Universitätsklinikum Freiburg, Hermann-Herder Str 11, 79104 Freiburg, Germany (zsolt.ruzsics@uniklinik-freiburg.de, jonas.fuchs@uniklinik-freiburg.de);

[5] Division of Immunology, Department of Medicine 3, FAU, Glückstraße 6, 91054 Erlangen, Germany (hans-martin.jaeck@fau.de);

[6] Drug Development Division, Infectious Disease Research, Southern Research, 431 Aviation Way, Frederick, Maryland 21701, USA (rptak@southernresearch.org)

[7] Immunic AG, Lochhamer Schlag 21, 82166 Gräfelfing, Germany (andreas.muehler@imux.com, evelyn.peelen@imux.com, manfred.groeppel@imux.com, daniel.vitt@imux.com, hella.kohlhof@imux.com).

[&] Both authors contributed equally to this study.

* Correspondence: manfred.marschall@fau.de; Tel.: +49 9131 8526089

Supplementary Figures

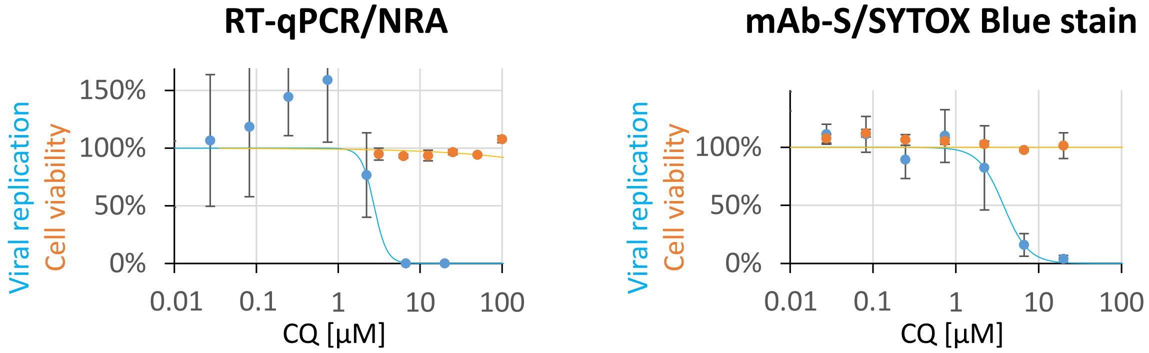

EC_{50}	2.7 ± 0.9 µM
CC_{50}	>100 µM
SI	>37

EC_{50}	3.8 ± 1.7 µM
CC_{50}	>20 µM
SI	>5

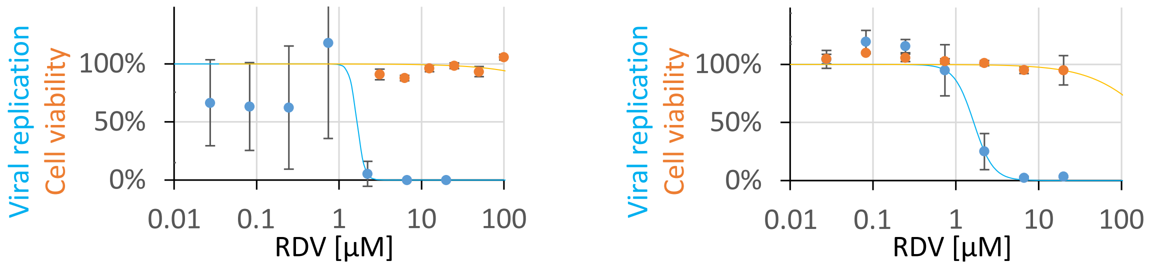

EC_{50}	1.7 ± 1.0 µM
CC_{50}	>100 µM
SI	>59

EC_{50}	1.7 ± 0.5 µM
CC_{50}	>20 µM
SI	>12

Figure S1. Antiviral activity and cytotoxicity determinations of reference drugs. Vero 76 cells were infected with severe acute respiratory syndrome coronavirus type 2 (SARS-CoV-2) and a multiplicity of infection (MOI) of 0.002 in presence of chloroquine (CQ) or remdesivir (RDV) at indicated concentrations. Viral replication was determined by RT-qPCR and cell viability by Neutral Red assay (NRA) (left panels). Additionally, fixed cells were analyzed for viral replication by detection of the viral S protein and cell numbers were quantitated by SYTOX Blue staining to detect drug-induced cell loss (right panels). Data points represent mean values ±SD of quadruplicate (RT-qPCR, mAb-S and SYTOX Blue staining) or triplicate (NRA) determinations. Logistic curve fitting was performed to visualize dose responses and for calculation of the EC_{50} values. EC_{50}, half-maximal effective concentration; CC_{50}, half-maximal cytotoxic concentration; SI, selectivity index CC_{50}/EC_{50}.

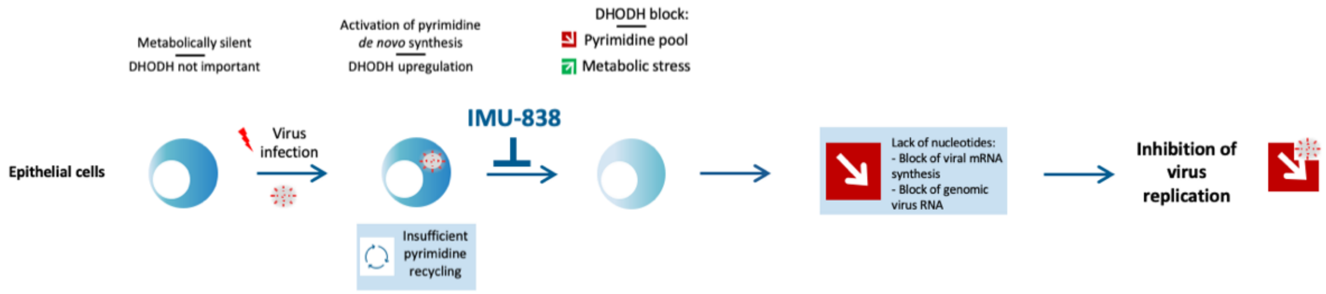

Figure S2. Schematic depiction of the mode of inhibitory activities of dihydroorotate dehydrogenase (DHODH)-directed drugs. Normal epithelial cells, i.e. not activated through specific stimuli and not virus-infected, are metabolically silent, so that DHODH activity may transiently only play a subordinate role. These cells are able to maintain their constant, but low-level need for pyrimidines mainly through the metabolic recycling pathway. Upon virus infection, however, the massive onset of viral gene expression and replication increases the demand for pyrimidines, which cannot be sustained by the pyrimidine recycling pathway alone anymore. Hence, viral infection strongly induces the pyrimidine *de novo* synthesis pathway involving the enzyme DHODH as a major, rate-limiting regulator. Thus, the treatment of virus-infected cells with IMU-838, or related DHODH inhibitory drugs, can lead to a critical inhibition of the required upregulation of DHODH activity and *de novo* pyrimidine synthesis.

Supplementary Methods

Methods for comparative antiviral analysis of human pathogenic viruses

For the use in human cytomegalovirus (HCMV) green fluorescent protein (GFP)-based replication assays, primary human foreskin fibroblasts (HFFs) were cultivated in 12-well plates (2.25 x 10^5 cells/well), infected with HCMV AD169-GFP (MOI 0.1–0.25, i.e. GFP-FU/mL according to 10-25% GFP-forming units detectable after 7 days) and treated with antiviral drugs added immediately after infection. At 7 days post infection (p.i.), cells were lysed and lysates were subjected to automated GFP quantification using a Victor 1420 Multilabel Counter (Perkin Elmer, Germany). All infections were performed in duplicate; GFP quantitations were performed in quadruplicate. Standard plaque reduction assays and reporter-based antiviral assays for the viruses tested were performed under previously established conditions [1-3]. Cytotoxicity was additionally assessed by incubating compounds at the concentrations of 0–10 μM on HFF monolayers for routine monitoring under the light microscope.

For hepatitis C virus (HCV), a Huh7 human hepatoma cell line containing a HCV subgenomic replicon of genotype 1b with a stable luciferase (Luc) reporter and three cell culture-adaptive mutations (Luc-ubi-neo ET, under license from Ralf Bartenschlager and Apath, LLC) was used [4]. The Luc-ubi-neo ET cell line was grown in Dulbecco's modified Eagle medium (DMEM), 10% fetal bovine serum (FBS), 100 units/mL penicillin, 100 μg/mL streptomycin, 2.0 mM L-glutamine and 250 μg/mL G418 in a 5% CO_2 incubator at 37 °C. The assay medium for anti-HCV evaluation was DMEM with 5% FBS, 100 units/mL penicillin, 100 μg/mL streptomycin and 2 mM L-glutamine. Cells were plated in 96-well plates (Costar) at 1.5 x 10^4 cells per well. The next day, vidofludimus (0–30 μM) or the positive control, recombinant interferon (IFN)α-2b (0–2 IU/mL), was added to the wells starting from a 40 mM stock and diluting it in medium to make 6 half-log serial dilutions. After 24 h incubation, the cells were processed and values of EC_{50} (50% inhibitory concentration), CC_{50} (50% cell viability concentration) and SI_{50} (selectivity index CC_{50}/EC_{50}) were calculated. HCV replicon levels were assessed with the replicon-derived luciferase activity as readout (SteadyGlo® Luciferase Assay System, Promega, Madison, WI, USA). The cytotoxic concentrations of drug-reducing cell numbers were assessed by the CytoTox-1 cell proliferation assay (Promega) according to the manufacturer's protocol.

For human immunodeficiency virus type 1 (HIV-1), fresh human blood was obtained commercially from Biological Specialty Corporation (Colmar, PA, USA). The clinical virus isolate HIV-$1_{91US005}$ (CCR5-tropic, subtype B) was obtained from the National Institutes of Health (NIH) acquired immunodeficiency syndrome (AIDS) Reagent Program (Division of AIDS, NIAID, NIH courtesy of Dr. Beatrice Hahn and the DAIDS). A low passage stock of this virus was prepared using fresh human PBMCs and stored in liquid nitrogen. A pre-titered virus aliquot was thawed rapidly to room temperature immediately before use. Fresh human PBMCs, seronegative for HIV and hepatitis B virus (HBV), were isolated from screened donors. Platelets were removed by low-speed centrifugation. PBMCs were isolated by density centrifugation for 30 min at 600 x g using Lymphocyte Separation Medium (LSM; Cellgro® Mediatech, Inc., VA, USA). Cells were cultivated in RPMI 1640 supplemented with 15 % FBS, 100 units/mL penicillin, 100 μg/mL streptomycin, and 2 mM L-glutamine, 4 μg/mL phytohemagglutinin (PHA, Sigma, St. Louis, MO, USA). After 48-72 h at 37 °C, cells were resuspended in fresh medium with 100 U/mL penicillin, 100 μg/mL streptomycin, 2 mM L-glutamine, 10 μg/mL gentamycin and 20 U/mL recombinant human IL-2 (R&D Systems Inc., Minneapolis, MN, USA). PBMCs were maintained for a maximum of 2 weeks in this medium with biweekly medium changes until used. For the assay, PHA-stimulated cells from at least two donors were pooled to minimize the interindividual donor variability. Stock virus was used for infection at predetermined dilutions (MOI approx. 0.1), so that vidofludimus-mediated virus inhibition and cytotoxicity could be determined (positive control azidothymidine/zidovudine, AZT). High-test concentrations of 100 μM and 1 μM of both vidofludimus and AZT, respectively, were initially used, followed by eight additional serial half-log dilutions, in a concentration range of 100–0.01 μM, or 1–0.1 nM, respectively. After 7 days, supernatants were collected for analysis of viral reverse transcriptase (RT) activity. Following removal

of supernatant samples, compound cytotoxicity was measured by addition of MTS to the plates and residual cell viability was measured (CellTiter 96 Reagent, Promega). To this end, MTS was incubated for 4-6 h at 37 °C before samples were spectrophotometrically analyzed at 490/650 nm with a Molecular Devices SpectraMaxPlus plate reader. Wells were also examined microscopically to ensure that no morphological cell abnormalities were noted. A microtiter plate-based RT reaction was performed [5], for which the RT reaction buffer was prepared freshly (125 µl 1.0 M EGTA, 125 µl dH_2O, 125 µl 20% TritonX100, 50 µl 1.0 M Tris pH 7.4, 50 µl 1.0 M DTT, and 40 µl 1.0 M $MgCl_2$). The final reaction mixture was prepared by combining one volume of ^{3}H-TTP (1 mCi/mL, NEN) and four volumes of dH_2O, 2.5 parts poly rA:oligo dT stock (Pharmacia) with 2.5 volumes of reaction buffer. This reaction mixture was incubated with virus-containing supernatants at a 1:1.5 ratio, and plates were incubated at 37 °C for 60 min. Following incubation, the reaction volume was spotted onto DE81 filter- mats (Wallac), followed by washing and drying of the filter mats, so that incorporated radioactivity (counts per min, CPM) could be quantitated using standard liquid scintillation techniques. In addition, viral p24 antigen was measured using an ELISA kit (XpressBio Life Science Products, Frederick, ML, USA) according to the manufacturer's instructions. Data were collected by spectrophotometric analysis at 450 nm using a Molecular Devices SpectraMaxPlus plate reader. Finally, p24 levels were calculated from the optical density values using the Molecular Devices SOFTmax Pro software package.

Supplementary References

1. Chou, S.; Marousek, G.; Auerochs, S.; Stamminger, T.; Milbradt, J.; Marschall, M. The unique antiviral activity of artesunate is broadly effective against human cytomegaloviruses including therapy-resistant mutants. *Antiviral research* **2011**, *92*, 364-368, doi:10.1016/j.antiviral.2011.07.018.
2. Marschall, M.; Freitag, M.; Weiler, S.; Sorg, G.; Stamminger, T. Recombinant green fluorescent protein-expressing human cytomegalovirus as a tool for screening antiviral agents. *Antimicrobial agents and chemotherapy* **2000**, *44*, 1588-1597, doi:10.1128/aac.44.6.1588-1597.2000.
3. Rechter, S.; König, T.; Auerochs, S.; Thulke, S.; Walter, H.; Dörnenburg, H.; Walter, C.; Marschall, M. Antiviral activity of Arthrospira-derived spirulan-like substances. *Antiviral research* **2006**, *72*, 197-206, doi:10.1016/j.antiviral.2006.06.004.
4. Pietschmann, T.; Lohmann, V.; Kaul, A.; Krieger, N.; Rinck, G.; Rutter, G.; Strand, D.; Bartenschlager, R. Persistent and transient replication of full-length hepatitis C virus genomes in cell culture. *Journal of virology* **2002**, *76*, 4008-4021, doi:10.1128/jvi.76.8.4008-4021.2002.
5. Buckheit, R.W., Jr.; Swanstrom, R. Characterization of an HIV-1 isolate displaying an apparent absence of virion-associated reverse transcriptase activity. *AIDS research and human retroviruses* **1991**, *7*, 295-302, doi:10.1089/aid.1991.7.295.
